# Childhood Body Weight in Relation to Cause-Specific Mortality: 67 Year Follow-up of Participants in the 1947 Scottish Mental Survey

**DOI:** 10.1097/MD.0000000000002263

**Published:** 2016-02-12

**Authors:** George David Batty, Catherine M. Calvin, Caroline E. Brett, Iva Čukić, Ian J. Deary

**Affiliations:** From the Department of Epidemiology and Public Health, University College London (GDB), London; Centre for Cognitive Ageing and Cognitive Epidemiology (GDB, CMC, CEB, IČ, IJD); and Department of Psychology, University of Edinburgh (CMC, CEB, IČ, IJD), Edinburgh, UK.

## Abstract

The association between childhood body weight and adult health has been little-examined, and findings are inconsistent.

In a representative sample of the Scottish nation (the Scottish Mental Survey of 1947), we examined the association between body mass index measured at 11 years of age and future cause-specific mortality by age 77 years. In this cohort study, a maximum of 67 years of follow-up of 3839 study members gave rise to 1568 deaths (758 from cardiovascular disease, 610 from any malignancy). After adjustment for covariates, there was some evidence of a relation between elevated childhood body mass index and rates of mortality ascribed to all-causes (hazard ratio per 1 SD increase in body mass index; 95% confidence interval: 1.09; 1.03, 1.14), cardiovascular disease (1.09; 1.01, 1.17), all cancers combined (1.12; 1.03, 1.21), smoking-related cancers (1.13; 1.03, 1.25), and breast cancer in women (1.27; 1.04, 1.56).

In conclusion, we provide further observational evidence for the need for weight control measures in youth.

## INTRODUCTION

It is now well established that overweight and obesity in middle- and older-aged populations are associated with later health outcomes, including coronary heart disease,^1,2^ stroke,^1,2^ and selected cancers.^1,3^ With repeat cross-sectional surveys of child populations conducted over the last 3 decades showing a secular rise in the prevalence of overweight and obesity,^4^ research attention is also being focused on the long-term health impact of body weight measured earlier in the life course. Such relationships are biologically plausible. Body mass index (BMI) tracks, such that overweight and obese children are more likely to become overweight and obese adults.^5^ Together with the well-documented relationship between adult BMI and future chronic disease, it is likely then that childhood overweight may, directly or indirectly, exert such an influence on adult health. Second, in cross-sectional studies of children, obesity has been shown to be associated with raised blood pressure and cholesterol,^6^ both of which predict selected health endpoints, such as cardiovascular disease, in later life.^7,8^

To date, many of the studies of childhood BMI and adult health are based on the ascertainment, by self-report, of early life body weight made in middle- and older-aged people.^9,10^ This raises concerns regarding the validity of such distantly recalled information. With studies having both a prospective assessment of early weight and health surveillance over several decades being somewhat rare, the role of early life weight in the etiology of long-term chronic disease is therefore not fully understood.^11^ In the few studies with prospective measurement of weight in childhood, there is a suggestion that higher BMI tends to be related to an elevated risk of premature mortality^11^ and coronary heart disease^12^ several decades later. It is currently not possible to form clear conclusions about links with other common presentations of cardiovascular disease (eg, stroke, heart failure, and peripheral vascular disease) nor with site-specific malignancies (eg, breast, colorectal, and stomach).

Accordingly, using data from the Scottish Mental Survey of 1947 (SMS1947), a nationally representative cohort of men and women, we related a measurement of BMI when the study members were 11 years of age to various mortality outcomes up to 67 years later. We hypothesized that overweight in childhood would be related to a series of mortality outcomes in later life, particularly death from all-causes, cardiovascular disease, and stroke.

## MATERIALS AND METHODS

The objective of the SMS1947 was to measure the intelligence of every 11-year-old child in all Scottish schools on 4th June 1947.^13,14^ A total of 88% of children took part (N = 70,805).^13,14^ A subgroup of study members (the “30-Day Sample”; N = 5083 children), comprising children born on the 1st, 2nd (even numbered months only), and 3rd days of each month of 1936 were selected for further research participation. This group, which is the subject of long-term follow as described herein, was representative of the full SMS1947 in terms of sex, geographical location, size of family, and cognitive test score.^15^ Our revitalization of the SMS1947 as a cohort study was approved by the Scotland-A Research Ethics Committee, the National Health Service Scotland Privacy Advisory Committee, and the Confidentiality Advisory Group of the Health Research Authority.^16^

For each study member, a head teacher populated a questionnaire (known as the “Sociological Schedule”) pertaining to each pupil's physical attributes and socioeconomic circumstances, including: physical disability, father's occupational level, and the number of people in their dwelling and the number of rooms. Physical disability/illness was denoted by a history of congenital paralysis, unheralded paralysis, deafness, epilepsy, chorea, defective vision, deafness, encephalitis, epilepsy, meningitis, or endocrine defects.^16^ Room occupancy was computed by dividing the number of people living in the dwelling by the number of rooms. The father's or main guardian's occupation was coded into 1 of 5 social class categorizations,^17^ ranging from professional (highest prestige) to unskilled. Height (inches) and weight (stone/pounds) were directly measured, and conversion to metric units allowed us to compute BMI using the standard formulae (weight [kg]/height^2^[m^2^]).

### Mortality Ascertainment

We electronically traced study members resident in Scotland using the National Health Service Central Register (NHSCR), with those who had migrated to England and Wales being located using the Medical Research Information Service Integrated Database and Administration System. The linkage covered the period 1st June 1947 to 1st October 2014 for Scotland-based study members (1st March 2014 for those resident in England and Wales).^18^ Causes of death were coded according to the International Classification of Disease (version 9^19^ or 10^20^).

### Statistical Analyses

Among the initial group of 5083 study participants in the 30 day sample, 4768 (94%) were traced. Exclusion of people with missing data for BMI, covariates, or cause of death resulted in an analytical sample of 3839 (1941 males and 1898 females). There was essentially no difference in BMI between individuals in the analytical sample (16.7 kg/m^2^) and those excluded (16.8 kg/m^2^; *P*-value for difference = 0.25). Cox proportional hazards regression analyses,^21^ with calendar time as the time scale, were used to compute hazard ratios with accompanying 95% confidence intervals to estimate the relationship between childhood BMI and later risk of cause-specific mortality. Study members were censored at age at death, or age by the end of the follow-up period – which ever occurred first. In preliminary analyses conducted separately for males and females there was no evidence that gender modified the relation between BMI and the causes of death reported herein, so allowing us to pool the data and sex-adjust the effect estimates. Using multivariable models, we also adjusted effect estimates for a series of other covariates which could plausibly be linked to both BMI and outcome: room occupancy, father's SES, height (all indicators of socioeconomic status), and physical disability. All analyses were undertaken using SPSS Statistics 21.

## RESULTS

A maximum of 67 years of follow-up of the 3839 study members gave rise to 1568 deaths (758 from cardiovascular disease, 610 from any malignancy). In Table 1, we present the association of childhood BMI with mortality from all causes and various cardiovascular disease presentations. There was evidence of a positive relation between weight and total mortality, such that elevated mortality rates were apparent in the higher weight groups. Similar patterns of association and magnitude were apparent when both mortality due to cardiovascular disease and coronary heart disease were the outcomes of interest, suggesting that these relations were partially generating the BMI–total mortality gradient. Adding covariates to the statistical models had very little impact on the strength of these hazard ratios in any of these analyses. There was no clear evidence that early life BMI was related to the other mortality endpoints in Table 1, those being myocardial infarction, all strokes, peripheral vascular disease, and heart failure.

**TABLE 1:**
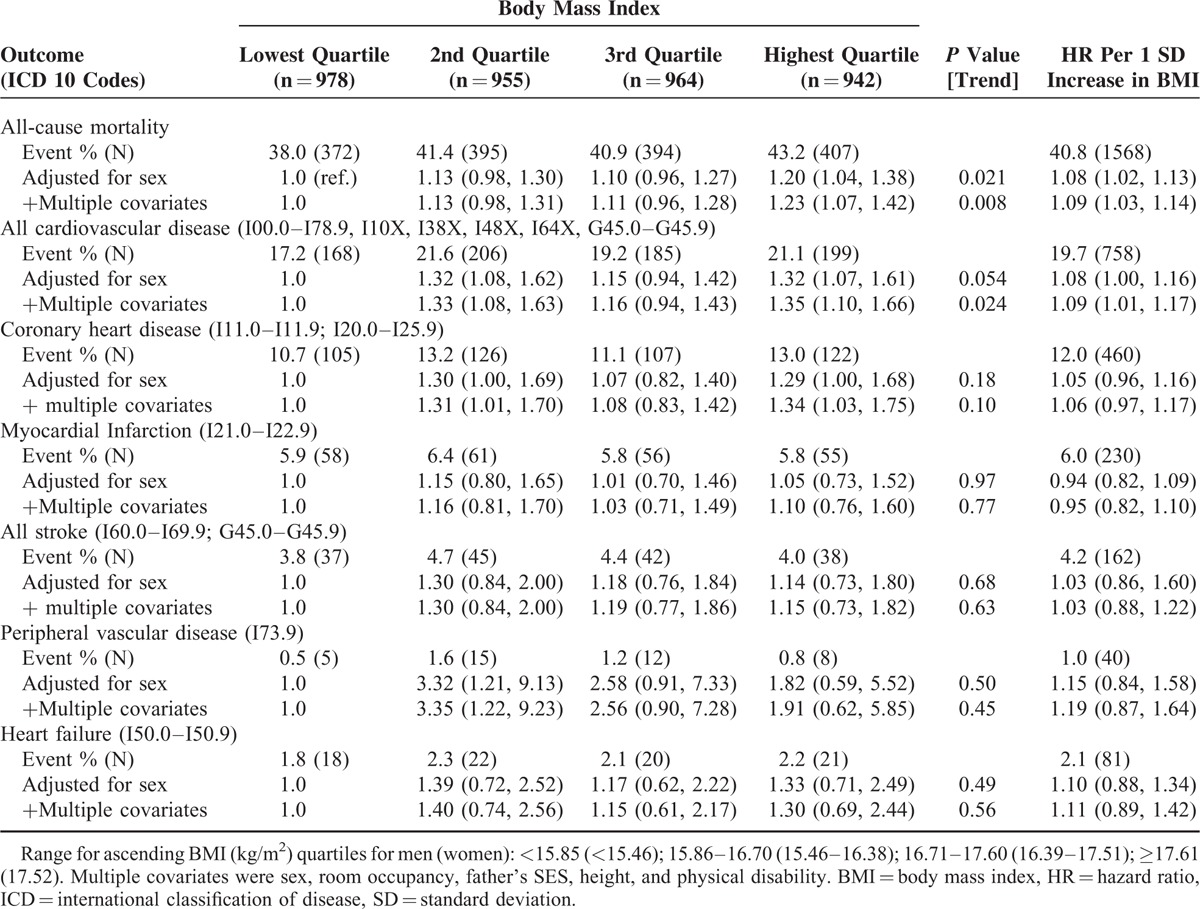
Hazard Ratios (95% Confidence Intervals) for Childhood Body Mass Index in Relation to Cardiovascular Disease Mortality – 67 Year Follow-up of Participants in the 1947 Scottish Mental Survey (N = 3839)

The results of the analyses for childhood BMI and subsequent risk of cancer mortality are depicted in Table 2. There was a positive relation between childhood BMI and the rate of death from all malignancies combined; this appeared to be partially generated by a similar pattern of association for smoking-related cancers. Further disaggregation into specific anatomical sites resulted in there being some evidence of a link between higher BMI and cancer of the lung (a component of smoking-related causes) and breast, although some of these analyses were underpowered judging by both the absolute number of deaths and the width of some confidence intervals.

**TABLE 2:**
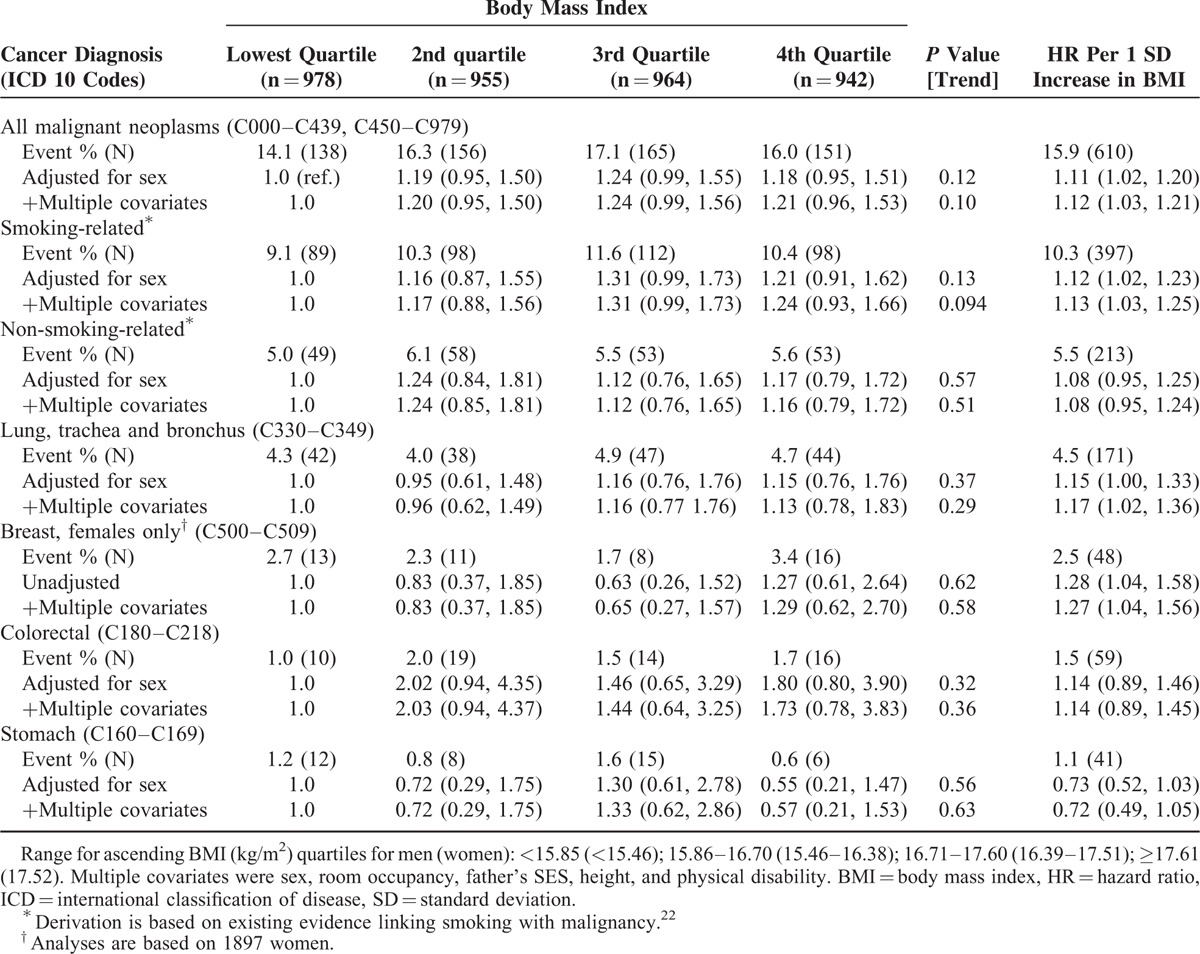
Hazard Ratios (95% Confidence Intervals) for Childhood Body Mass Index in Relation to Selected Site-Specific Cancer Mortality – 67 Year Follow-Up of Participants in the 1947 Scottish Mental Survey (N = 3839)

## DISCUSSION

Taking the results from the present study together, the main finding was that there was evidence that higher weight at 11 years of age was associated with elevated rates of several mortality outcomes later in life. Our results accord with some existing findings. Total mortality, among the better explored health outcomes in relation to pre-adult weight, typically reveals a direct association.^11^ In a systematic review of coronary heart disease risk in which the authors stratified studies into age at BMI assessment, for the age range most relevant to our own (7–17 years; 7 studies), the aggregated association (hazard ratio per 1 SD increase in BMI; 95% CI: 1.09; 1.00, 1.07) resembles the effect estimate reported herein (1.06; 0.97, 1.17).^23^ As described, in cross-sectional studies of children, obesity has been shown to be associated with blood pressure and cholesterol, both of which predict selected health endpoints, such as cardiovascular disease, in later life. This is therefore 1 mechanism via which higher BMI could confer an increased risk of cardiovascular disease. We did not, however, have these necessary data with which to test this assertion.

In an evaluation of 8 studies featuring stroke as the outcome of interest, 3 found no relationship as we did.^11^ To the best of our knowledge, ours is the 1st examination of the link between childhood BMI and other presentations of cardiovascular disease, particularly heart failure and peripheral vascular disease. With cancer being less common than cardiovascular disease, studies in the context of a prospective measurement of early life weight are particularly rare. Results for total cancers are mixed, as they are for individual sites.^11^ We found a positive relation for BMI and smoking-related cancers that was weaker when cancers unrelated to smoking was the endpoint of interest. The absence of data on smoking in the present study is unlikely to be crucial in this context because smoking has a BMI-lowering effect so could only explain a negative association between BMI and cancer, not a positive one.

### Study Strengths and Limitations

The present study has a series of strengths relative to some others in this field including the prospective measurement of BMI, analyses of an array of chronic diseases outcomes to enhance insights into specificity, and the generalizability of results from a study based on a nationally representative sample. In particular, examining the relation of overweight in childhood and early adult groups who, relative to middle- and older-age groups, are comparatively disease-free, has the advantage of minimizing the problem of confounding such that poor health can lead to both weight loss and generate an increased risk of mortality. With the underlying BMI-mortality relation being positive, studies such as ours are more likely to yield the most accurate estimate of this gradient. Our study is not without its weaknesses, however. As described, BMI tracks across the life course such that overweight children have an increased likelihood of becoming overweight adults.^5^ It is therefore plausible that the apparent increased rates of selected mortality endpoints may be ascribed to overweight in adulthood rather than childhood. Although this does not necessarily diminish the importance of childhood overweight – it may simply point to a mechanistic pathway – we had no repeat measurement of BMI with which to test this hypothesis.

In conclusion, we found that higher childhood body weight was related to a series of mortality outcomes in later adulthood. These findings of a long-term impact of childhood overweight are additional to the now well-documented acute detrimental effects and point to the importance of weight control measures in younger age groups.
